# Blood lipids, lipid-regulatory medications, and risk of bladder cancer: a Mendelian randomization study

**DOI:** 10.3389/fnut.2023.992608

**Published:** 2023-12-22

**Authors:** Zhang Cheng, Fangdie Ye, Yingchun Liang, Chenyang Xu, Zheyu Zhang, Yuxi Ou, Xinan Chen, Xiyu Dai, Zezhong Mou, Weijian Li, Yiling Chen, Quan Zhou, Lujia Zou, Shanhua Mao, Haowen Jiang

**Affiliations:** ^1^Department of Urology, Huashan Hospital, Fudan University, Shanghai, China; ^2^Fudan Institute of Urology, Huashan Hospital, Fudan University, Shanghai, China; ^3^National Clinical Research Center for Aging and Medicine, Huashan Hospital, Fudan University, Shanghai, China

**Keywords:** blood lipids, lipid-regulatory medications, bladder cancer, Mendelian randomization, UK Biobank, FinnGen

## Abstract

**Background:**

The influences of blood lipids and lipid-regulatory medications on the risk of bladder cancer have long been suspected, and previous findings remain controversial. We aimed to assess the causality between blood lipids or lipid-regulatory medications and bladder cancer susceptibility by means of a comprehensive Mendelian Randomization (MR) study.

**Methods:**

Genetic proxies from genome-wide association studies (GWAS) of four blood lipid traits and lipid-lowering variants in genes encoding the targets of lipid-regulatory medications were employed. The largest ever GWAS data of blood lipids and bladder cancer involving up to 440,546 and 205,771 individuals of European ancestry were extracted from UK Biobank and FinnGen Project Round 6, respectively. A two-sample bidirectional MR study was performed using the inverse variance weighted as the main method. The heterogeneity, horizontal pleiotropy, MR Steiger, and leave-one-out analyses were also conducted as sensitivity tests.

**Results:**

There was indicative evidence that genetically predicted low-density lipoprotein cholesterol (LDL-C) affected bladder cancer susceptibility based on 146 single nucleotide polymorphisms (SNPs) with an odds ratio (OR) of 0.776 (95% confidence interval [CI] = 0.625–0.965, *p* = 0.022). However, this result became non-significant after two SNPs that possibly drove the effect were removed as demonstrated by leave-one-out analysis. The reversed MR analysis suggested that bladder cancer could not affect serum lipid levels. No causal relationship was found between the lipid-lowering effect of lipid-regulatory medications (fibrates, probucol, statins, ezetimibe, proprotein convertase subtilisin/kexin type 9 [PCSK9] inhibitors, and evinacumab) and the risk of bladder cancer. No heterogeneity or pleiotropy was found (all *p* > 0.05).

**Conclusion:**

This MR study revealed for the first time, using the most recent and comprehensive GWAS data to date, that genetically predicted total cholesterol (TC) and the lipid-lowering effect of lipid-regulatory medications had no causal association with bladder cancer susceptibility. We also verified claims from early studies that low-density lipoprotein cholesterol (HDL-C), LDL-C, and triglyceride (TG) are not related to bladder cancer susceptibility either. The current study indicated that lipid metabolism may not be as important in the tumorigenesis of bladder cancer as previously believed.

## Introduction

According to the estimation of the International Agency for Research on Cancer and the American Cancer Society, there were approximately 573,000 newly diagnosed cases and 212,000 disease-related deaths of bladder cancer around the globe in 2020 alone, making it the tenth most common cancer worldwide and the most likely encountered malignancy for urologists (1). Although the incidence of bladder cancer is far higher in men, for whom it is the sixth most prevalent cancer, the past 2 decades have witnessed a rise in women’s tobacco consumption, which has in turn contributed to the increased trend in incidence among women, while the trend for men has remained stable (2, 3). Non-muscle invasive bladder cancer is characterized by a high risk of recurrence after the initial transurethral resection and requires frequent imaging examinations or even, when necessary, a second transurethral cystoscopy, albeit being confined to the mucosa or submucosa at the early stage (4). Eventually, approximately 30% of non-muscle invasive bladder cancer will progress into muscle-invasive one during the course of the disease (5). While radical cystectomy with postoperative platinum-based chemotherapy has been the first-line treatment for advanced-stage patients for decades, the illness remains refractory and often metastasizes to adjacent organs, lungs, or the liver (2). The emergence of immunotherapy with checkpoint inhibitors has somewhat improved the prognosis of terminal bladder cancer (6, 7). However, its response rate is still modest (8), not to mention the local and systemic side effects it brings about, resulting in treatment discontinuation (7). Therefore, primary prevention of bladder cancer is of crucial importance not only for patients themselves but also for the reduction of socio-economic burdens for families and society alike.

It is widely recognized that smoking is a key risk factor for bladder cancer (3) though other factors such as occupational exposure to aromatic amines and infection with *Schistosoma haematobium* may also be major contributors in certain populations (2, 9). The roles of dyslipidemia and lipid-regulating therapy in the etiology of bladder cancer have long been suspected. A Chinese case–control study recruiting 972 patients with pathologically diagnosed urothelial carcinoma of the bladder and 1,098 cancer-free controls discovered that hypertriglyceridemia was positively related to bladder cancer (10), while a previous MR study using limited SNPs showed no correlations between TG, LDL-C or HDL-C, and bladder cancer susceptibility (11). The different and even controversial outcomes among studies may possibly attribute to the size of study populations, statistical power, and biases. Thus, higher-quality, well-designed research with a broader subject scale and fewer biases of any kind is urgently required to address this issue.

Mendelian randomization, a novel statistical strategy that makes use of genetic variants associated with the exposure of interest as instruments, has been increasingly applied to estimate the causal effects of risk factors on the outcome (12). Due to the random allocation of genes during conception and gamete formation, MR is less susceptible to confounding influences that emerge over one’s lifespan. As a result, it overcomes the drawbacks of conventional observational studies (13). Moreover, genetic variants adjacent to or within the genes that code for drug targets can regulate their expression and, therefore, affect the activities of pharmacological targets. These genetic variants can anticipate and reflect the therapeutic effects of drugs on individuals as previously described in antihypertensive medicine (14). To the best of our knowledge, there has only been one MR study that employed merely one SNP named rs12916 as a surrogate for 3-hydroxy-3-methylglutaryl-coenzyme A reductase (HMG-CoA reductase, *HMGCR*), the target of statins, to investigate the association between statins use and the risk of bladder cancer (11).

Herein, we aimed to perform a two-sample, bidirectional MR analysis to assess the causal effects of genetically predicted blood lipids and genetic proxies for lipid-regulatory medications on the risk of bladder cancer in a comprehensive and reliable manner using the largest GWAS data for blood lipids and bladder cancer to date.

## Methods

### Data sources

The biggest publicly available genome-wide association study for blood lipids to date was acquired from the UK Biobank^1^ and IEU-GWAS summary data^2^ in order to enlarge the sample size and avoid biases to the greatest extent. The details of the study protocol have been published in their previous study (15). For four representatives of circulating lipids, namely, HDL-C, LDL-C, TC, and TG, 403,943, 440,546, 115,078, and 115,078 European participants were enlisted, respectively (16). Since all the participants from UK Biobank went through the same standard blood lipid testing procedures with laboratory results being continuous variables, the effects of any change in blood lipids on the outcome were represented as one standard deviation.

GWAS summary association statistics for bladder cancer were downloaded from the FinnGen Project.^3^ It is a gigantic initiative first launched in 2017, encompassing everything from genetic information to digital health care data, much to the UK Biobank database. In-depth quality control procedures were clarified by its earlier studies (17). The most recent data from Round 6 was released on January 24, 2022, which contained 1,701 cases of malignant neoplasm of the bladder and 204,070 controls (all types of cancers were excluded from controls) of European ancestry. While the UK Biobank datasets of blood lipids were large enough to support sufficient statistical power, a modest degree of statistical power was expected for the outcome due to the relatively small number of cases of bladder cancer in the FinnGen Project compared to that of controls (18).

In general, all the data utilized in this study is open to the public. Informed consent was obtained from all participants in the original genome-wide association studies which were authorized by their corresponding ethics committee. Information on data sources is listed in Supplementary File 1.

### Instrumental variables identification

SNPs with *p*-values less than 5 × 10^−8^ were selected from the UK Biobank GWAS for the four blood lipid traits. Those who were palindromic or in linkage disequilibrium (LD, threshold: *r*^2^ < 0.001, kb = 10,000) based on the 1,000 Genomes Project reference panel were discarded. By identifying confounder-related SNPs in an online phenome-wide association study database^4^ and the Phenotype Scanner^5^, SNPs closely related to the potential confounders, including smoking, body mass index, waist-to-hip ratio, and type 2 diabetes mellitus, were also excluded. The remaining SNPs were correspondingly chosen as instrumental variables (IVs) for their genetic associations with HDL-C, LDL-C, TC, and TG.

As for the causal effect of pharmacological interventions on bladder cancer, we adopted genetic IVs as proxies for the LDL-C lowering effect of lipid-regulatory medications commonly used in clinical practice. The following drugs with indications concerning lipid metabolism were not recruited for either exerting multisystemic effects (e.g., niacin) or are rarely prescribed by physicians (e.g., Mipomersen, a binder to the mRNA encoding apolipoprotein B, is only available to patients with homozygous familial hypercholesterolemia under a restricted clinical program due to its liver toxicity). Then, we searched for the validated targets implicated in the lipid metabolic process of these drugs at the DrugBank^6^ and collected their SNPs recorded by GeneCards.^7^ Similarly, SNPs that were genetically correlated with LDL-C (*p* < 5 × 10^−8^) and underwent LD-clumping (threshold *r*^2^ < 0.4, kb =10,000) were identified as final IVs for each class of lipid-regulatory medication upon the exclusion of confounder-related SNPs mentioned above. Such a relatively loose threshold allowed for an increase in the number of IVs, the proportion of variance explained, and hence the statistical power. In addition, we also set a stricter LD-clumping threshold of *r*^2^ < 0.001.

### Statistical analysis

After harmonizing the alleles for consistency, MR analyses were conducted for estimations of causal effects by five complementary methods: the inverse variance weighted (IVW), MR-Egger, weighted median, weighted mode, and simple mode, with the IVW being the main approach established on the assumption that instrumental variables affected the outcome solely through the exposure of interest (19). When only one SNP was available, the Wald ratio was calculated instead. The estimates of median-based approaches remain robust even when half of the SNPs are weak instruments (20). However, as these methods were still susceptible to pleiotropy, we examined the intercept of MR-Egger regression and employed MR-Pleiotropy Residual Sum and Outlier methods (MR-PRESSO) for detection of horizontal pleiotropy despite having minimized confounding biases (21, 22). To assess for the underlying heterogeneity, Cochran’s Q-value was estimated by the IVW and MR-Egger (23). Leave-one-out analysis, where an instrumental SNP was removed one at a time to analyze the causal effects of the remaining SNPs on the outcome, was also performed as a sensitivity analysis to determine if the MR results were driven by any particular SNP. Furthermore, we performed the MR Steiger test of directionality to judge whether the results yielded were in the same direction as our hypothesis. The causal direction was evaluated as true if all of the IVs together explained more variance in the exposure than the outcome (24). The *F* statistics were generated for all the exposure in case of weak instrument biases caused by SNPs with *F* statistics less than 10 (25).

**Figure 1:**
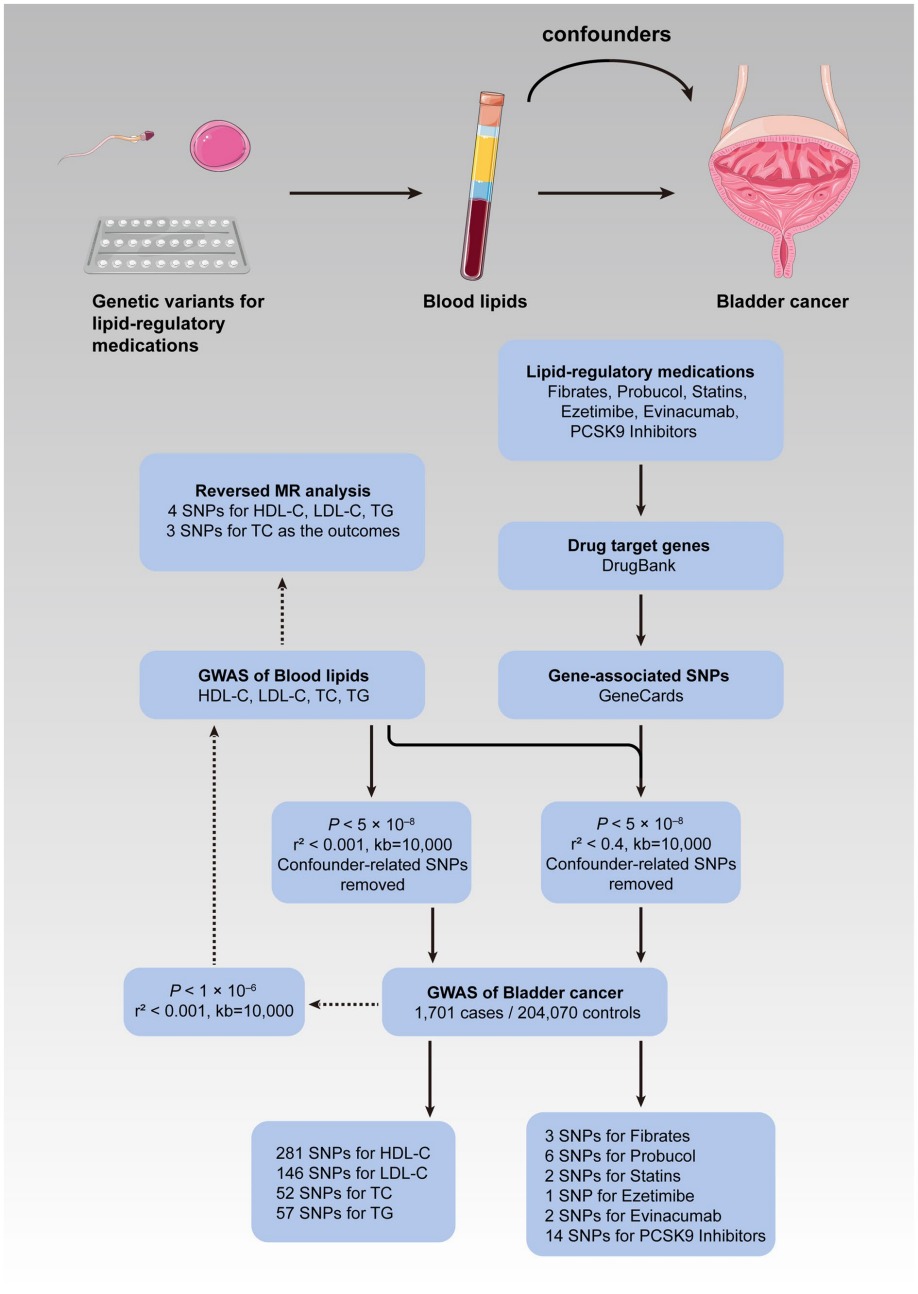
Flow diagram of the current MR study. The upper panel illustrated the conceptual framework for the MR analysis of lipid-regulatory medications and the risk of bladder cancer, while the lower panel showed the entire design of the current study. MR, Mendelian randomization; GWAS, genome-wide association study; SNPs, single nucleotide polymorphisms; HDL-C, high-density lipoprotein cholesterol; LDL-C, low-density lipoprotein cholesterol; TC, total cholesterol; TG, triglyceride; PCSK9, proprotein convertase subtilisin/kexin type 9.

The current MR study consisted of two stages. First of all, the causal effects of the four blood lipid traits on the risk of bladder cancer were investigated, followed by a reversed MR analysis where bladder cancer was treated as the exposure, while the blood lipids as the outcomes in order to rule out reverse causality. We loosened the standard of significance for IVs to *p* < 1 × 10^−6^ in the reversed procedure so as to enhance statistical power and lower the false-negative rate. Subsequently, a second MR analysis involving lipid-lowering therapy and bladder cancer was performed. The correlation was considered statistically significant after the Bonferroni correction for multiple hypothesis testing for four blood lipid traits (*p* < 0.0125) and six types of medications (*p* < 0.0083). A *p*-value greater than the adjusted cutoff while less than 0.05 was considered indicative of evidence for probable causality. The statistical significance of the analyses above was indicated as a two-tailed *p*-value less than 0.05. All of the analyses were carried out in R (version 4.0.3) with “TwoSampleMR” and “MR-PRESSO” being the primary packages used (26). Figure 1 depicts the conceptual framework for the MR analysis of lipid-regulatory medications and the risk of bladder cancer as well as the entire design of the current study.

## Results

### Genetically determined blood lipids and the risk of bladder cancer

**Figure 2:**
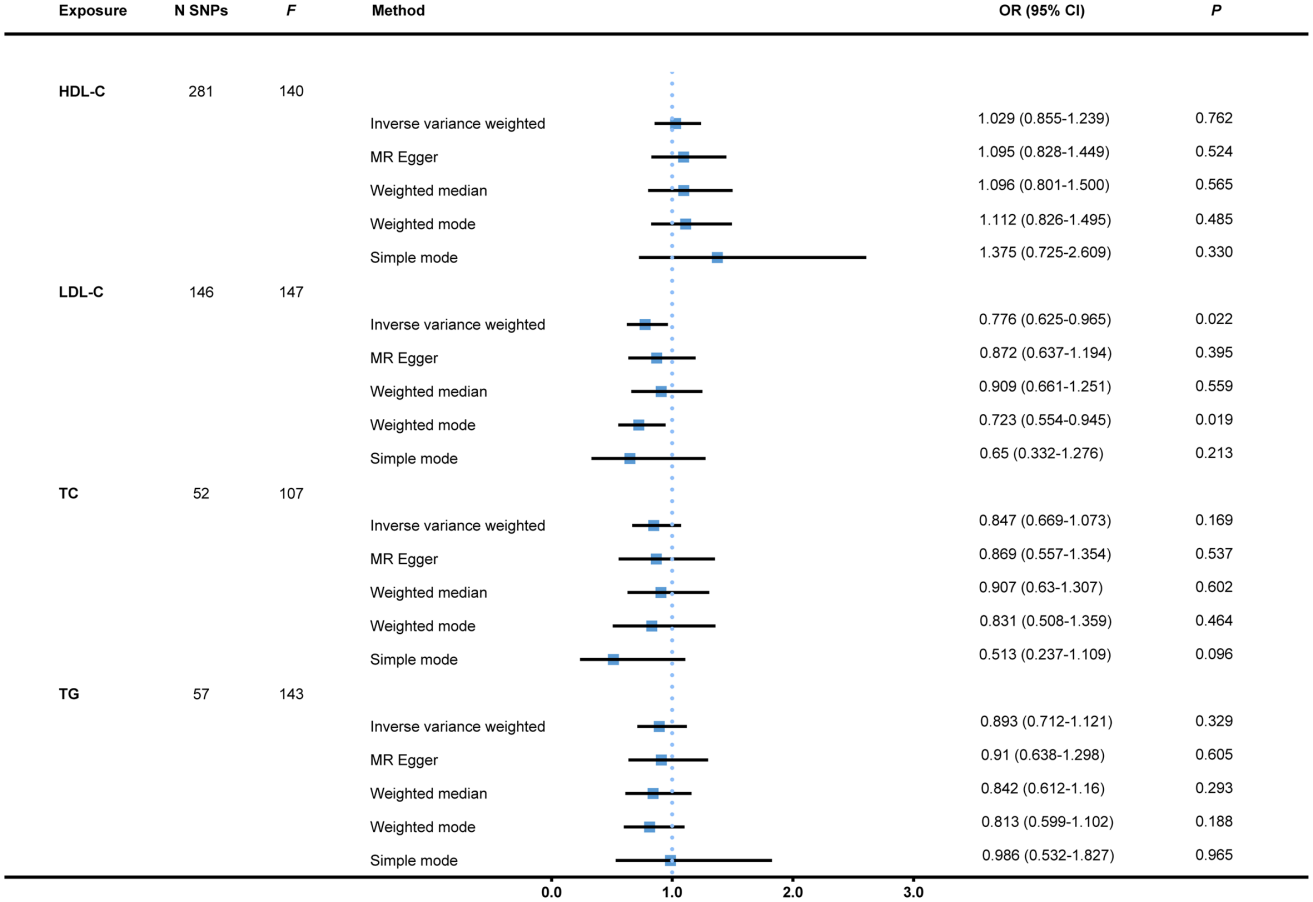
MR analysis of causal effects of genetically predicted blood lipids on the risk of bladder cancer. Genome-wide significantly associated (*p* < 5 × 10^−8^) independent (LD-clumping *r*^2^ < 0.001, kb = 10,000) SNPs were selected as instrumental variables. However, the causal effect of LDL-C on bladder cancer susceptibility became non-significant with an OR of 0.832 (95% CI = 0.659–1.050, *p* = 0.121, as not shown in the figure) after two SNPs possibly driving the effect as demonstrated by the leave-one-out analysis were removed. MR, Mendelian randomization; N SNPs, number of single nucleotide polymorphisms; HDL-C, high-density lipoprotein cholesterol; LDL-C, low-density lipoprotein cholesterol; TC, total cholesterol; TG, triglyceride; OR, Odds ratio; CI, confidence interval; LD, linkage disequilibrium.

To start with, we examined the causal relations between genetically determined blood lipids and the risk of bladder cancer. After the initial filtering and complete exclusion of confounder-related SNPs, a total of 281, 146, 52, and 57 instrumental variants in close connection with HDL-C, LDL-C, TC, and TG, respectively, were identified. All the 536 IVs are displayed in Supplementary File 2. There was no causal effect of genetically predicted HDL-C, TC, or TG on the risk of bladder cancer with all *p*-values >0.05. Nevertheless, the result indicated a causal link of suggestive evidence between LDL-C and bladder cancer via the IVW approach with an OR of 0.776 (95% CI = 0.625–0.965, *p* = 0.022). This was further corroborated by the weighted mode (OR = 0.723, 95% CI = 0.554–0.945, *p* = 0.019; Figure 2). There were two single SNPs closely linked to LDL-C that plausibly drove the connection as demonstrated by the leave-one-out analysis. With these two instrumental variants removed, the result became insufficient to draw a conclusion of causality between LDL-C and bladder cancer susceptibility with an OR of 0.832 (95% CI = 0.659–1.050, *p* = 0.121). There was neither evidence of heterogeneity (IVW and MR-Egger) nor horizontal pleiotropy (MR-Egger intercept and MR-PRESSO) in all of the analyses (Table 1). Scatter plots and leave-one-out plots are presented in Supplementary File 3.

**Table 1:** Heterogeneity and pleiotropy tests with bladder cancer as the outcome.

Exposure	N SNPs	Heterogeneity analysis				Pleiotropy analysis	
		Method	Q	df	*p*-value	Egger intercept (*p* value)	MR-PRESSO *P*
HDL-C	281	MR-Egger	314.79	279	0.069	−2.49 × 10^−3^ (0.560)	0.087
		IVW	315.17	280	0.073		
LDL-C	146	MR-Egger	163.22	144	0.130	−5.76 × 10^−3^ (0.319)	0.136
		IVW	164.35	145	0.130		
TC	52	MR-Egger	43.81	50	0.719	−1.57 × 10^−3^ (0.896)	0.745
		IVW	43.83	51	0.752		
TG	57	MR-Egger	71.35	55	0.068	−1.52 × 10^−3^ (0.893)	0.084
		IVW	71.38	56	0.081		
Fibrates	3	MR-Egger	0.05	1	0.827	1.71 × 10^−2^ (0.778)	/
		IVW	0.18	2	0.914		
Probucol	6	MR-Egger	1.43	4	0.840	9.11 × 10^−2^ (0.354)	0.787
		IVW	2.52	5	0.773		
Statins	2	IVW	0.33	1	0.565	/	/
Ezetimibe	1	/				/	/
PCSK9 inhibitor	14	MR-Egger	6.65	12	0.880	−3.99 × 10^−3^ (0.837)	0.924
		IVW	6.69	13	0.917		
Evinacumab	2	IVW	0.14	1	0.708	/	/

SNP, single nucleotide polymorphisms; HDL-C, high-density lipoprotein cholesterol; LDL-C, low-density lipoprotein cholesterol; df, degree of freedom; IVW, inverse variance weighted; PCSK9, Proprotein convertase subtilisin/kexin type 9. The SNPs of lipid-regulatory medications were selected by a relatively loose LD threshold (*r*^2^ < 0.4, kb = 10,000).

**Figure 3:**
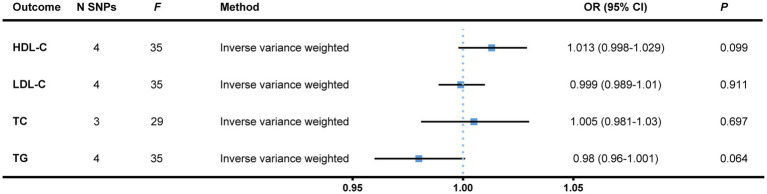
Reversed MR analysis with bladder cancer treated as the exposure, and blood lipids, the outcomes. Independent (LD-clumping *r*^2^ < 0.001, kb = 10,000) SNPs of bladder cancer from the FinnGen Project with a relatively loose standard of genetical significance (*p* < 1 × 10^−6^) were selected as instrumental variables. The IVW method was employed to calculate causal effects. MR, Mendelian randomization; N SNPs, number of single nucleotide polymorphisms; HDL-C, high-density lipoprotein cholesterol; LDL-C, low-density lipoprotein cholesterol; TC, total cholesterol; TG, triglyceride; OR, odds ratio; CI, confidence interval; LD, linkage disequilibrium; IVW, inverse variance weighted.

In the reversed MR stage, we gained 141 SNPs whose *p*-values were less than 1 × 10^−6^ out of a total of 16,355,128 SNPs from Round 6 data of the FinnGen Project, among which we finally confirmed four independent SNPs as genetic instruments for bladder cancer as the exposure. No significant results were detected albeit the heterogeneity arose when total serum cholesterol was treated as the endpoint. The findings remained the same after a single SNP analysis and the removal of an outlier (Figure 3 and Supplementary File 4). All of the heterogeneity and pleiotropy tests came out negative (Supplementary File 5).

### Genetic proxies for lipid-regulatory medications and the risk of bladder cancer

**Figure 4:**
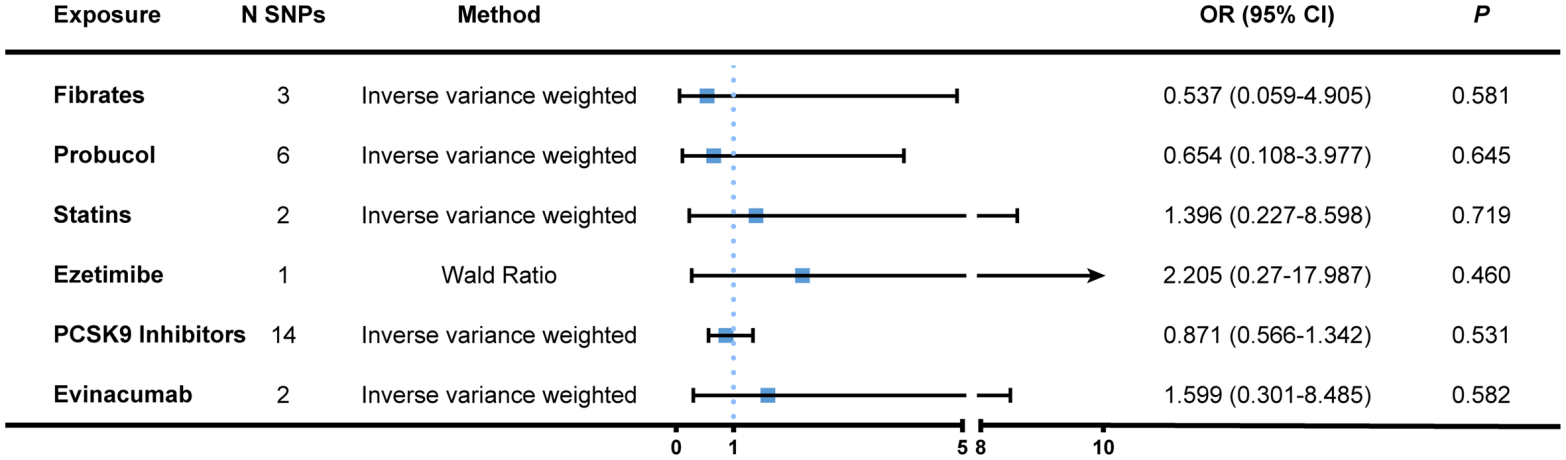
MR association between genetically proxied lipid-regulatory medications and the risk of bladder cancer. Genome-wide significantly associated (*p* < 5 × 10^−8^) SNPs of LDL-C with a relatively loose standard of LD-clumping (*r*^2^ < 0.4, kb = 10,000) were selected as instrumental variables. The IVW and Wald ratio (when only one SNP was available) was employed as the main approach to calculate causal effects. Complete results were displayed in Supplementary File 5. MR, Mendelian randomization; N SNPs, number of single nucleotide polymorphisms; OR, odds ratio; CI, confidence interval; PCSK9, proprotein convertase subtilisin/kexin type 9; LD, linkage disequilibrium; IVW, inverse variance weighted.

Second, we chose SNPs that genetically predicted the lipid-lowering effect of genes that encode targets aimed by lipid-regulatory medications as genetic proxies. After eliminating confounder-related SNPs, a total of 28 IVs were identified, including three for fibrates, six for probucol, two for statins, one for ezetimibe, 14 for PCSK9 inhibitors (alirocumab, evolocumab, and inclisiran), as well as two for evinacumab (Supplementary File 6). Nonetheless, we failed to acquire the proxies for adenosine triphosphate-citrate lyase (ACLY), the target of bempedoic acid. All IVs had *F* statistics greater than 10. There was no causation found between lipid-lowering therapy and the risk of bladder cancer (Figure 4 and Supplementary File 7). The Cochran’s Q statistics of both the IVW and MR-Egger methods showed little heterogeneity among genetic variants. Neither did the pleiotropy tests exhibit notable horizontal pleiotropy (Table 1).

An additional investigation into the causal effect of lipid-lowering intervention on the risk of bladder cancer using a more stringent LD criterion of *r*^2^ < 0.001 displayed comparable results (Supplementary File 8) though there was no suitable genetic instrument for statins under this LD threshold. The reason why we did not mix multiple types of lipid-regulatory medications to conduct a multivariant MR was that these drugs are usually administered alone or in combination with therapies treated for other chronic conditions. All of the aforementioned analyses passed the MR Steiger test, indicating that the directionality was correct (Supplementary File 9).

## Discussion

In general, an MR study was based on three core assumptions. First, the SNPs chosen as IVs are closely related to the exposure of interest. Second, the IVs have no relationship with any confounding factors. Third, the risk of the outcome is not affected except by the exposure (12, 13, 27). Realizing only the first assumption could be fully satisfied, we managed to circumvent biases to the largest extent by excluding SNPs closely associated with confounders, i.e., smoking, body mass index, waist-to-hip ratio, and type 2 diabetes mellitus.

In the current two-sample MR study, we used genetic variants as proxies for four circulating lipids and the lipid-lowering effect of six lipid-modulatory medications to probe into their causal effects on the risk of bladder cancer. The result of LDL-C initially showed suggestive evidence of the risk of bladder cancer; however, it became non-significant after omitting two SNPs identified as driving SNPs by the leave-one-out analysis. Although the analysis yielded little evidence of causal impacts for HDL-C, LDL-C, and TG on bladder cancer, which was consistent with a previous MR study (11), it was, to the best of our knowledge, the first MR study to shed light on the causal effects of TC as well as various classes of lipid-regulatory medications on bladder cancer susceptibility.

A case–control study in 2015 enrolling 2,070 Chinese people, including 972 newly diagnosed urothelial carcinoma of the bladder and 1,098 cancer-free controls, revealed a positive association between hypertriglyceridemia and bladder cancer (adjusted OR 1.254, 95% CI 1.020–1.542, *p* = 0.032) (10). In addition, triglycerides were found to be positively linked with the overall bladder cancer risk in men in a sex-stratified prospective study involving six European cohorts, regardless of whether the tumor was muscle invasive or not (hazard ratio [HR] = 1.17, 95% CI = 1.06–1.27). Teleka et al. also discovered in 2018 that an elevated level of triglycerides (HR = 1.30, 95% CI = 1.12–1.48) and cholesterol (HR = 1.14, 95% CI = 1.02–1.25) were associated with a higher incidence of non-muscle invasive bladder cancer among men (28). In 2018, however, Orho-Melander et al. observed no correlations between TG (OR = 0.96, 95% CI = 0.68–1.37), LDL-C (OR = 0.89, 95% CI = 0.65–1.22) or HDL-C (OR = 0.86, 95% CI = 0.64–1.16) and bladder cancer risk in a prior MR study, which used 26, 32, and 41 SNPs as proxies for three serum lipid characteristics, respectively (11). What we unraveled in our study verified the results of Orho-Melander’s with the most recent and comprehensive GWAS statistics available to date. The statistical power of our study was further enhanced by the enrollment of 1,701 patients with bladder malignant neoplasms as opposed to only 400 bladder cancer cases in Orho-Melander M’s MR investigation.

Statins, which inhibit the activity of HMGCR, are the most widely prescribed lipid-regulatory medications for the treatment of dyslipidemia, cardiovascular diseases, chronic kidney disease, and so on. A retrospective study found no link between statin use and the risk of bladder cancer despite the small number of participants enrolled (29). On behalf of statin use, only one SNP, rs12916 in the *HMGCR* gene, was employed in Orho–Menlander’s MR study and suggested no relation with bladder cancer risk (11). A more recent investigation found that 17,708 post-coronary syndrome patients, either randomized to the ezetimibe group, a cholesterol absorption inhibitor that blocks the sterol transporter Niemann-Pick C1-Like 1 (NPC1L1) on the intestinal villi of the enterocytes, or the matching placebo group, had similar incidences of malignancy and malignancy-related death, including bladder cancer (30).

There was very little research that focused on the influence of other lipid-regulatory medications or their targets on the risk of bladder cancer, let alone those using Mendelian Randomization. Hence, what we presented here was a novel study that not only probed into the causal effects of representative traits for blood lipids on the risk of bladder cancer through the largest cohorts to date but also for the first time elucidated the causal relationship between the lipid-lowering effect and bladder cancer susceptibility using genetically proxied lipid-modulatory medications. We thoroughly explored the targets of commonly prescribed lipid-regulatory drugs in clinical practice and strictly adhered to MR procedures by discarding confounding-related SNPs, applying sensitivity tests of heterogeneity, horizontal pleiotropy, leave-one-out analysis, and so forth.

The findings of this study shall be interpreted in light of its limitations. First, despite having access to the largest and latest GWAS data, the study could still be prone to weak instrument biases, particularly in the case of lipid-regulatory medications, which had a restricted number of genetic proxies available. Larger GWAS for both bladder cancer and diverse sorts of blood lipid characteristics with more reliable genetic tools may enable us to evaluate the causal effects more precisely. Second, due to the original GWAS statistics, we were unable to divide the cohorts by gender and make use of sex-specific genetic IVs for blood lipid traits and lipid-regulatory medications, which might not be a pivotal influencing factor in the results because a recently published gender-stratified GWAS covering 33 biomarkers from the UK Biobank discovered that the gender actually exerted a limited impact on the genetics of most traits (31). Similarly, as the outcome of this study, bladder cancer owns distinct types of histopathology, such as the most prevalent urothelial carcinoma, and the squamous carcinoma, which mainly occurred in sub-Saharan African populations, etc. Future GWAS with specific pathological types of bladder cancer are needed. Third, we selected genetic proxies for lipid-regulatory medications based on key MR assumptions. In other words, it reflected the life-long modulation of the lipid-lowering effect, making it impossible to estimate the effect on the risk of bladder cancer in the short run. Fourth, it has been reported that the gut microbiota might contribute to blood lipid fluctuation in the host as well (32), but the MR study focuses solely on the human genome to explore the causality. Fifth, the GWAS data analyzed in the present study came entirely from the European population, which could lead to biases based on race, geographic environment, and diet. We are looking forward to more GWAS with populations of diverse races in future as well as growing international cooperation to benefit the health of mankind through Mendelian randomization.

## Conclusion

In summary, using the latest and largest GWAS data to date, the current MR study discovered for the first time that genetically predicted TC and lipid-lowering effect of lipid-regulatory medications had no causal relationship with the risk of bladder cancer. We confirmed that HDL-C, LDL-C, and TG are not associated with bladder cancer susceptibility either. The results indicated that blood lipids may not play a crucial role in the tumorigenesis of bladder cancer and should be interpreted with caution. Further large-scale randomized controlled trials with various ethnic groups are warranted to validate our MR results.

## Data availability statement

The datasets presented in this study can be found in online repositories. The names of the repository/repositories and accession number(s) can be found in the article/Supplementary material.

## Author contributions

The study was conceived, designed, and analyzed by ZC. The manuscript was drafted by ZC and FY. HJ revised the manuscript and supervised the whole research. Others contributed to the summary of existing literature. All authors contributed to the article and approved the submitted version.

## Footnotes

^1^ https://www.ukbiobank.ac.uk/

^2^ https://gwas.mrcieu.ac.uk/

^3^ https://www.finngen.fi/en

^4^ https://gwas.mrcieu.ac.uk/phewas/

^5^ http://www.phenoscanner.medschl.cam.ac.uk/

^6^ https://go.drugbank.com/

^7^ https://www.genecards.org/
