# Supplementary material for: Blood lipids, lipid-regulatory medications, and risk of bladder cancer: a Mendelian randomization study
Source: Front Nutr. 2023 Dec 22;10:992608. doi: 10.3389/fnut.2023.992608 (PMC10768687; doi:10.3389/fnut.2023.992608)
Supplement: Supplementary file 1 [file Data_Sheet_1.PDF]

Supplementary File 5. The heterogeneity and pleiotropy tests of the reversed MR analysis with bladder cancer as the exposure

Outcome	N SNPs	Heterogeneity analysis				Pleiotropy analysis	
		Method	Q	df	*P* value	Egger intercept (*P* value)	MR-PRESSO *P*
HDL-C	4	MR-Egger	1.40	2	0.496	-4.70×10^{-2} (0.132)	0.115
		IVW	7.51	3	0.057		
LDL-C	4	MR-Egger	0.45	2	0.797	9.56×10^{-3} (0.691)	0.886
		IVW	0.66	3	0.881		
TC	3	MR-Egger	1.94	1	0.163	-1.88×10^{-2} (0.868)	/
		IVW	2.03	2	0.363		
TG	4	MR-Egger	0.81	2	0.668	3.16×10^{-2} (0.512)	0.095
		IVW	1.43	3	0.698		

MR: Mendelian randomization; N SNPs: Number of single nucleotide polymorphisms; HDL-C: High-density lipoprotein cholesterol; LDL-C: Low-density lipoprotein cholesterol; TC: Total cholesterol; TG: Total triglycerides; df: Degree of freedom; IVW: Inverse variance weighted
