# Supplementary material for: Blood lipids, lipid-regulatory medications, and risk of bladder cancer: a Mendelian randomization study
Source: Front Nutr. 2023 Dec 22;10:992608. doi: 10.3389/fnut.2023.992608 (PMC10768687; doi:10.3389/fnut.2023.992608)
Supplement: Supplementary file 2 [file Data_Sheet_2.PDF]

Supplementary File 6. The genetic proxies for lipid-lowering effect of lipid-regulatory medications

SNP	beta.exposure	se.exposure	pval.exposure	eaf.exposure	effect allele	other allele	target gene
**Fibrates**							
rs13076933	-0.020994	0.00239027	1.60E-18	0.259454	G	T	*PPARG*
rs147667955	-0.0782069	0.0123081	2.10E-10	0.007456	T	C	*PPARG*
rs2938388	0.0127517	0.00220252	7.10E-09	0.659553	G	A	*PPARG*
**Statins**							
rs17647994	-0.0350333	0.00500357	2.50E-12	0.045374	C	T	*HMGCR*
rs61602173	0.0532682	0.00334073	3.10E-57	0.108964	T	C	*HMGCR*
**Probucol**							
rs11791258	-0.0153344	0.00264184	6.50E-09	0.194301	A	G	*ABCA1*
rs12686004	-0.026899	0.00327027	1.90E-16	0.114891	A	G	*ABCA1*
rs13284054	-0.0280318	0.00322578	3.60E-18	0.124529	C	T	*ABCA1*
rs2254819	-0.0162494	0.0020911	7.80E-15	0.469064	C	T	*ABCA1*
rs2740488	-0.0252452	0.00236503	1.30E-26	0.265466	C	A	*ABCA1*
rs4149307	0.0218168	0.00287508	3.20E-14	0.155251	T	C	*ABCA1*
**Ezetimibe**							
rs2073547	0.0355498	0.00267287	2.30E-40	0.184007	G	A	*NPC1L1*
**PCSK9 Inhibitors**							
rs10888897	0.0293024	0.00212914	4.30E-43	0.606922	T	G	*PCSK9*

	rs11206517	0.0680285	0.00580615	1.00E-31	0.033149	G	T	*PCSK9*
	rs11591147	-0.348456	0.00793088	1.00E-200	0.017468	T	G	*PCSK9*
	rs11800243	-0.0309061	0.0051457	1.90E-09	0.042896	A	G	*PCSK9*
	rs11810371	-0.0294547	0.00507333	6.40E-09	0.043743	A	G	*PCSK9*
	rs12117661	-0.0471674	0.00242466	2.70E-84	0.246216	G	C	*PCSK9*
	rs12136600	-0.0430606	0.00415221	3.40E-25	0.068672	T	C	*PCSK9*
	rs12739394	-0.024023	0.0023194	3.90E-25	0.720521	G	C	*PCSK9*
	rs12739979	-0.0202563	0.00254032	1.50E-15	0.246521	T	C	*PCSK9*
	rs138483078	0.032716	0.00294516	1.10E-28	0.146789	A	G	*PCSK9*
	rs17111503	0.0406795	0.00235743	1.00E-66	0.268141	G	A	*PCSK9*
	rs287227	-0.0455567	0.00314027	1.10E-47	0.125262	T	G	*PCSK9*
	rs472495	0.0425743	0.00218093	7.30E-85	0.648959	T	G	*PCSK9*
	rs4927194	-0.047486	0.00671013	1.50E-12	0.024612	T	C	*PCSK9*
**Evinacumab**								
	rs1168128	0.0385467	0.00217832	4.50E-70	0.647045	G	C	*ANGPTL3*
	rs646179	-0.0241171	0.00214512	2.50E-29	0.60732	G	A	*ANGPTL3*

SNP: single nucleotide polymorphisms; se: standard error; eaf: effect allele frequency; *PPARG*: Peroxisome proliferator-activated receptor gamma; *HMGCR*: Hydroxy-methylglutaryl-CoA Reductase; *ABCA1*: ATP-binding cassette sub-family A member 1; *NPC1L1*: Niemann-Pick C1-like protein 1; *PCSK9*: Proprotein convertase subtilisin/kexin type 9; *ANGPTL3*: Angiopoietin Like 3
