# Supplementary material for: Blood lipids, lipid-regulatory medications, and risk of bladder cancer: a Mendelian randomization study
Source: Front Nutr. 2023 Dec 22;10:992608. doi: 10.3389/fnut.2023.992608 (PMC10768687; doi:10.3389/fnut.2023.992608)
Supplement: Supplementary file 3 [file Table_1.DOCX]

**Supplementary File 1.**

| IEU-GWAS ID | Trait | Year | Sample Size | N SNPs | Consortium | Population |
| --- | --- | --- | --- | --- | --- | --- |
| ieu-b-110 | LDL-C | 2020 | 440,546 | 12,321,875 | UKB | EUR |
| ieu-b-109 | HDL-C | 2020 | 403,943 | 12,321,875 | UKB | EUR |
| met-d-Total_C | TC | 2020 | 115,078 | 12,321,875 | UKB | EUR |
| met-d-Total_TG | TG | 2020 | 115,078 | 12,321,875 | UKB | EUR |
| / | malignant neoplasm of bladder | 2022 | 205,771 | 16,355,128 | FinnGen | EUR |

A summary of GWAS data used in the current Mendelian Randomization study

GWAS: Genome-wide association study; N SNPs: Number of single nucleotide polymorphisms

met-d datasets: Metabolic biomarkers in the UK Biobank measured by Nightingale Health 2020
