# Supplementary material for: Blood lipids, lipid-regulatory medications, and risk of bladder cancer: a Mendelian randomization study
Source: Front Nutr. 2023 Dec 22;10:992608. doi: 10.3389/fnut.2023.992608 (PMC10768687; doi:10.3389/fnut.2023.992608)

**Supplementary File 3.** The scatter and leave-one-out plots of MR analysis between blood lipids and bladder cancer risk.

The scatter plot of HDL-C


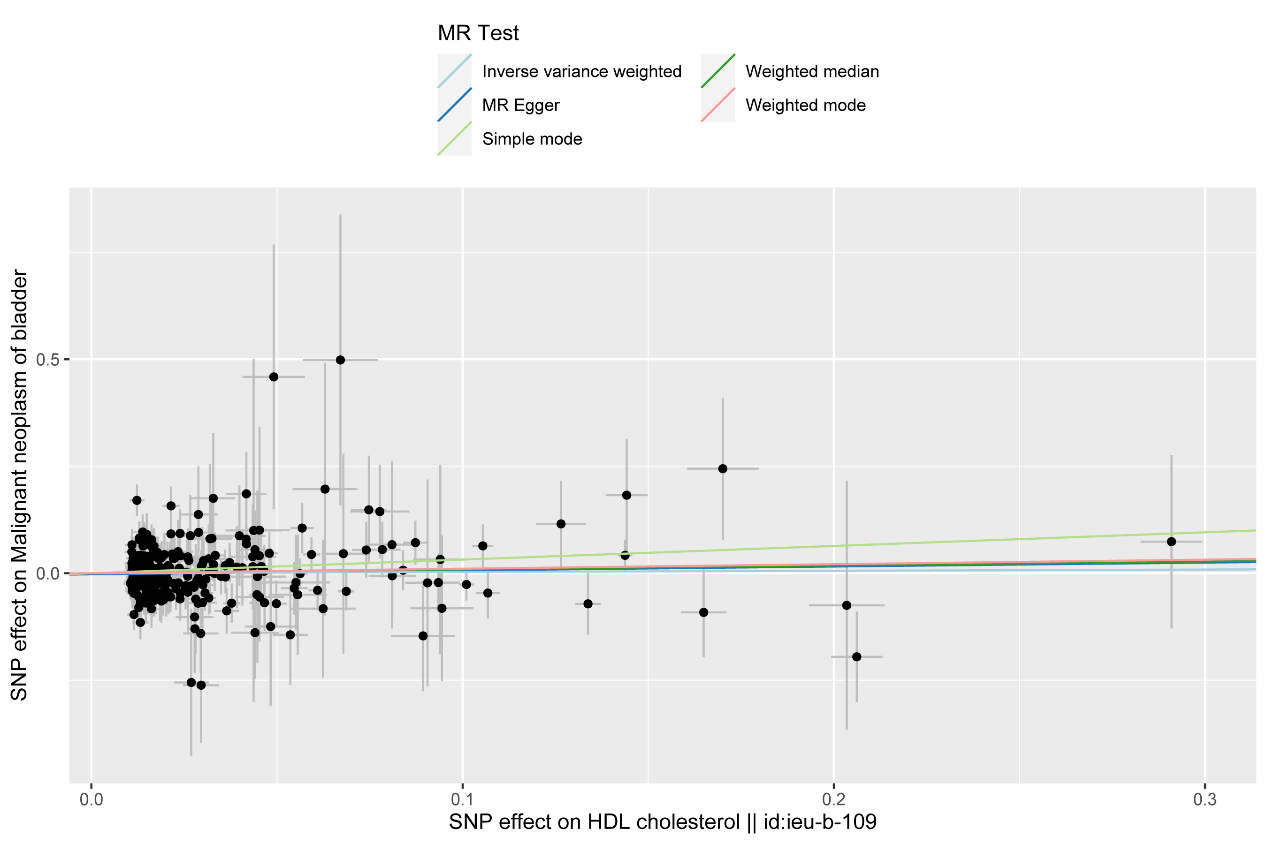


The scatter plot of LDL-C


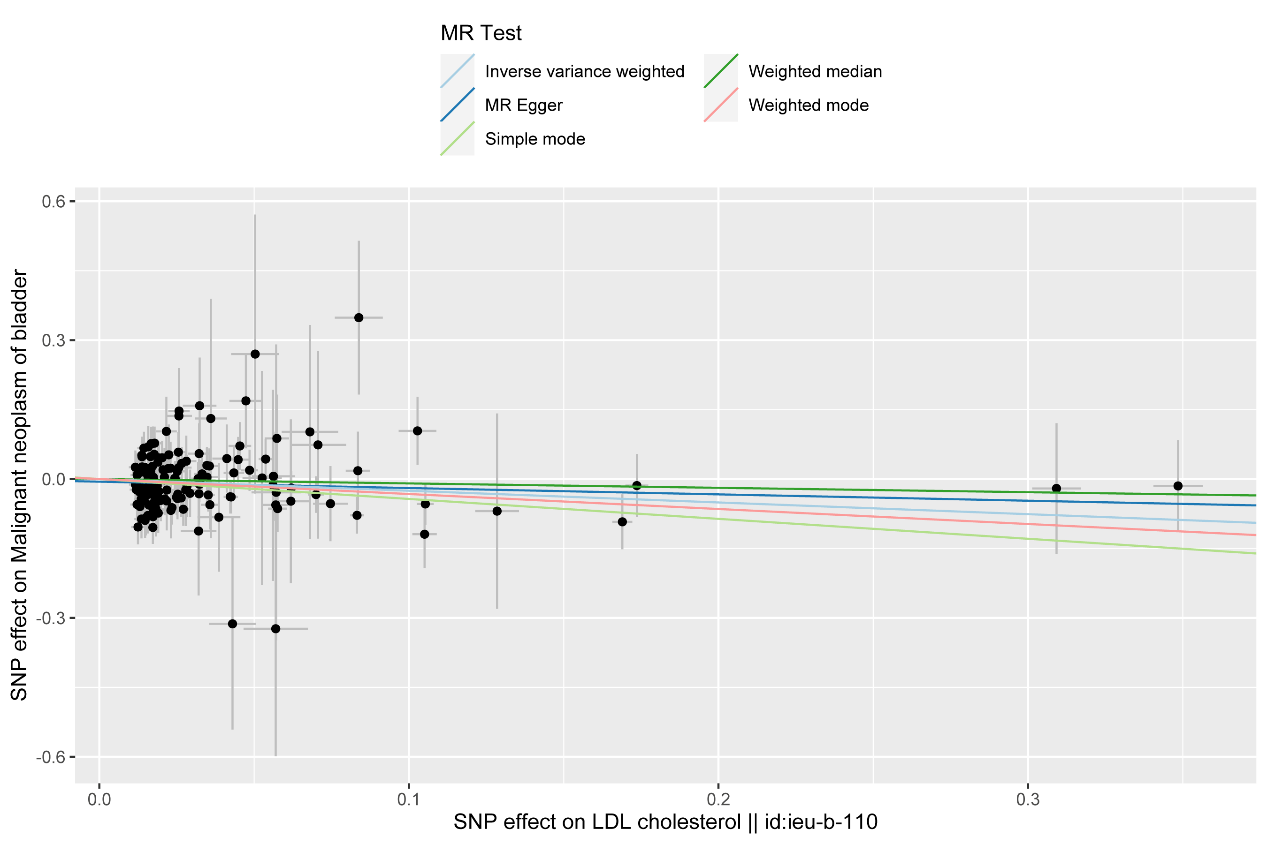


The scatter plot of TC


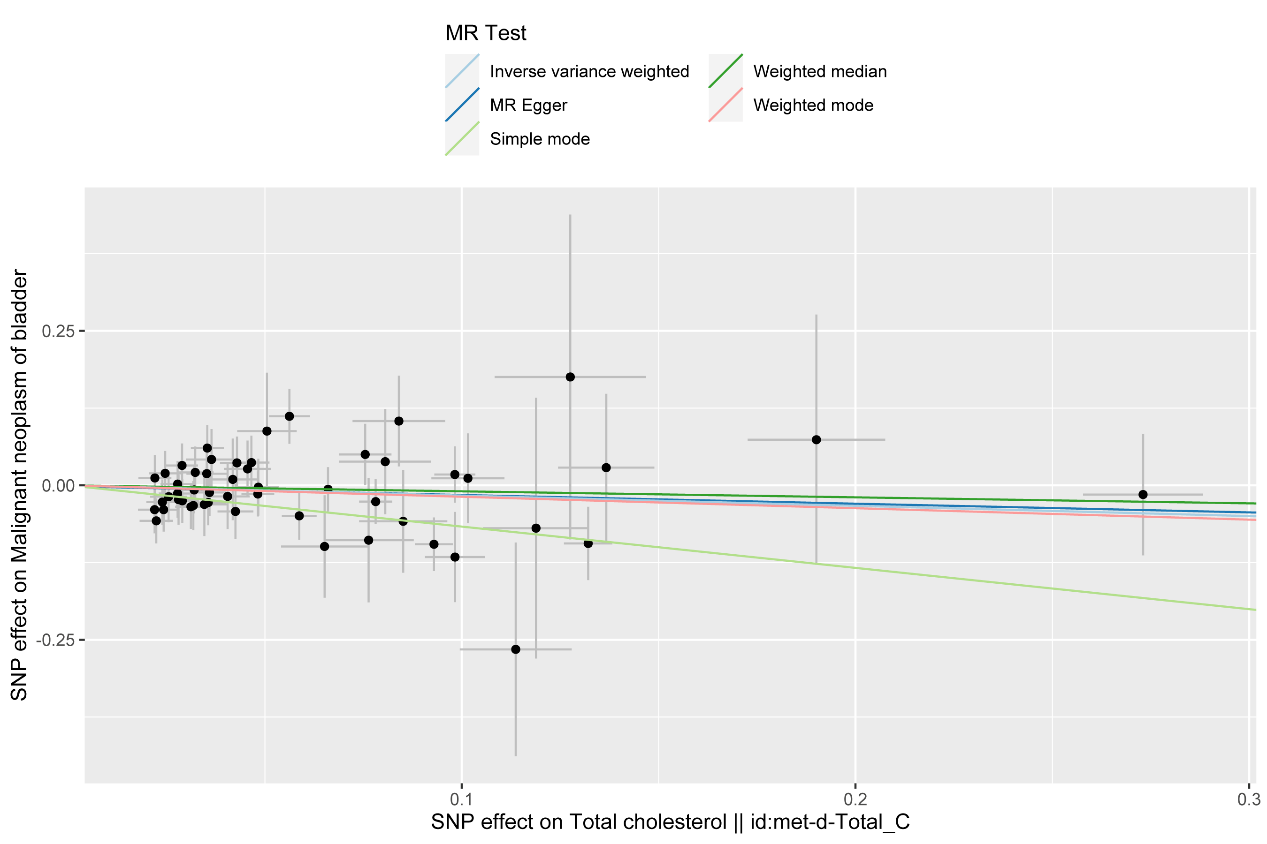


The scatter plot of TG


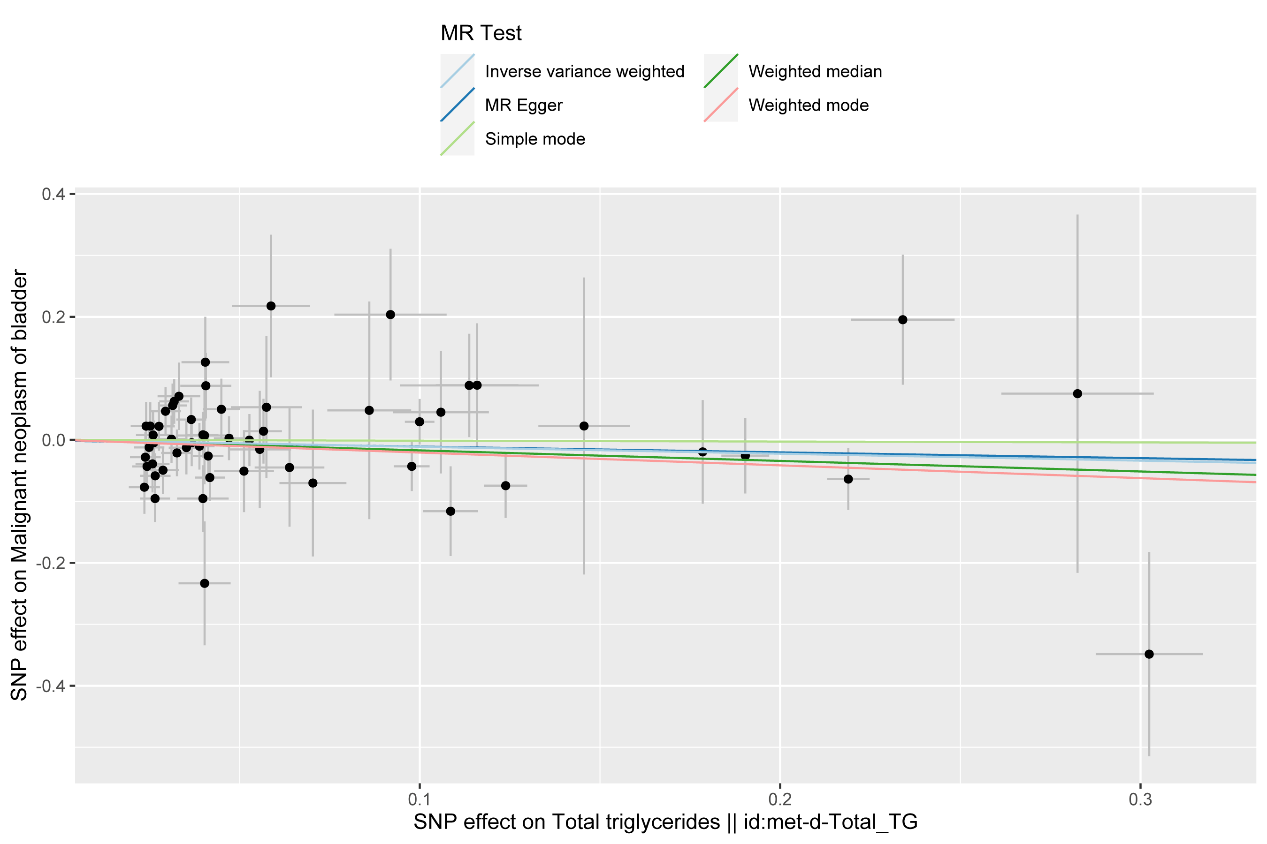


The leave-one-out plot of HDL-C


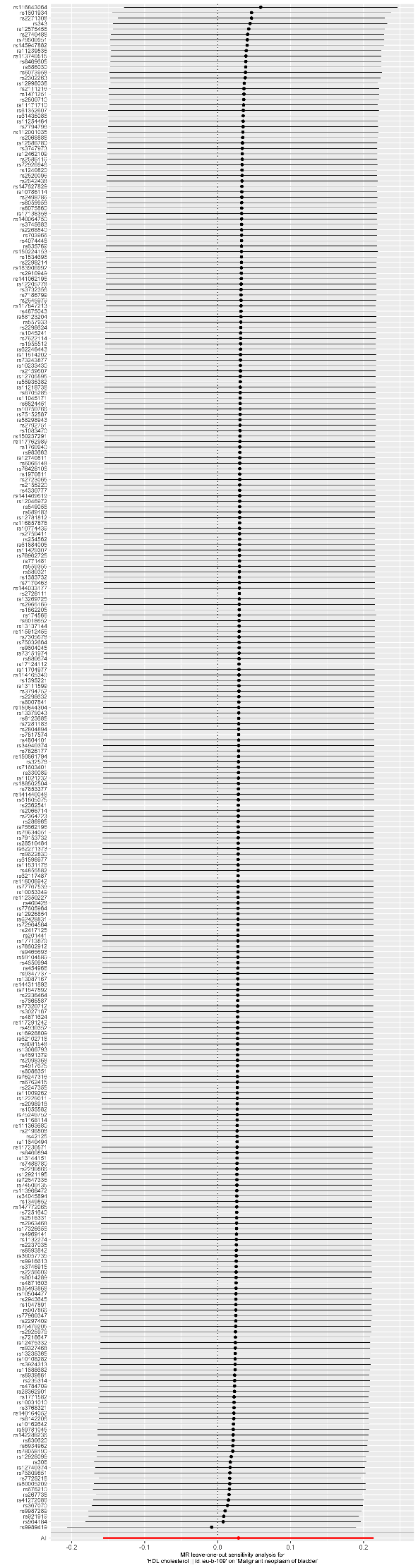


The leave-one-out plot of LDL-C (before removing the first two SNPs)


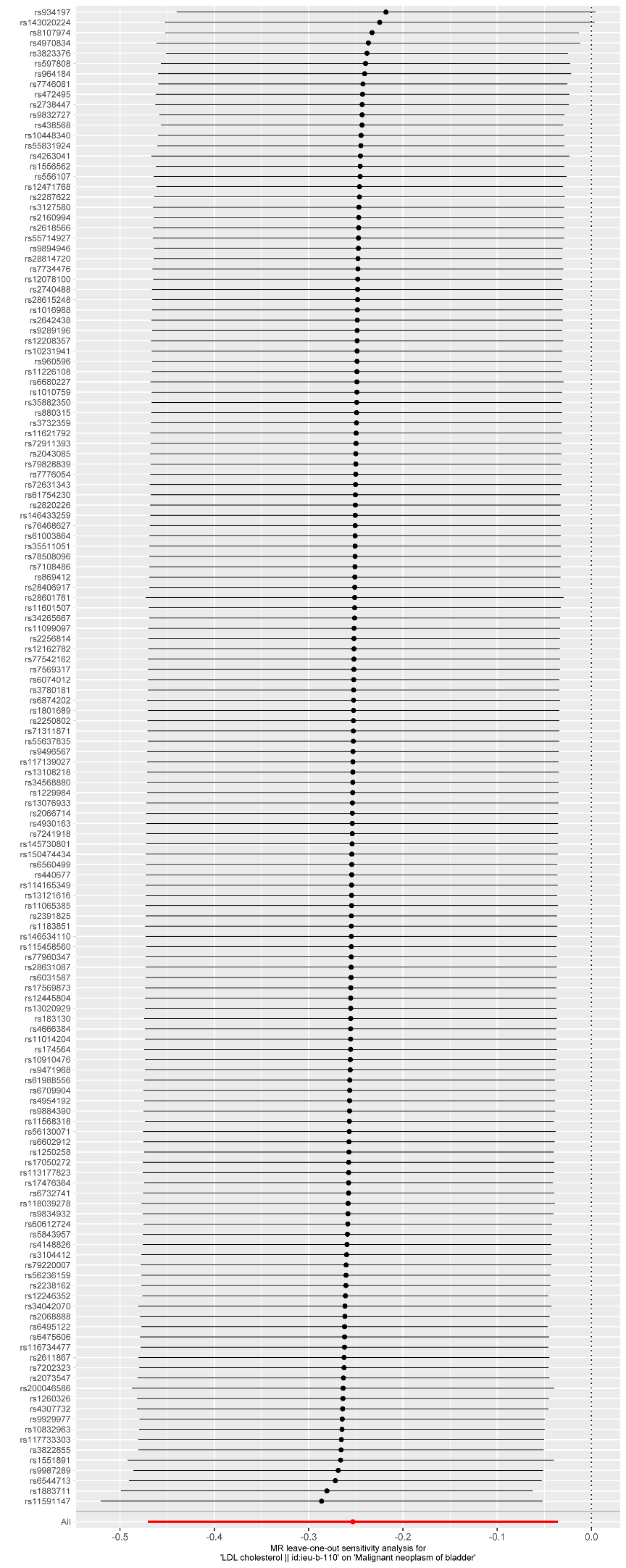


The leave-one-out plot of TC


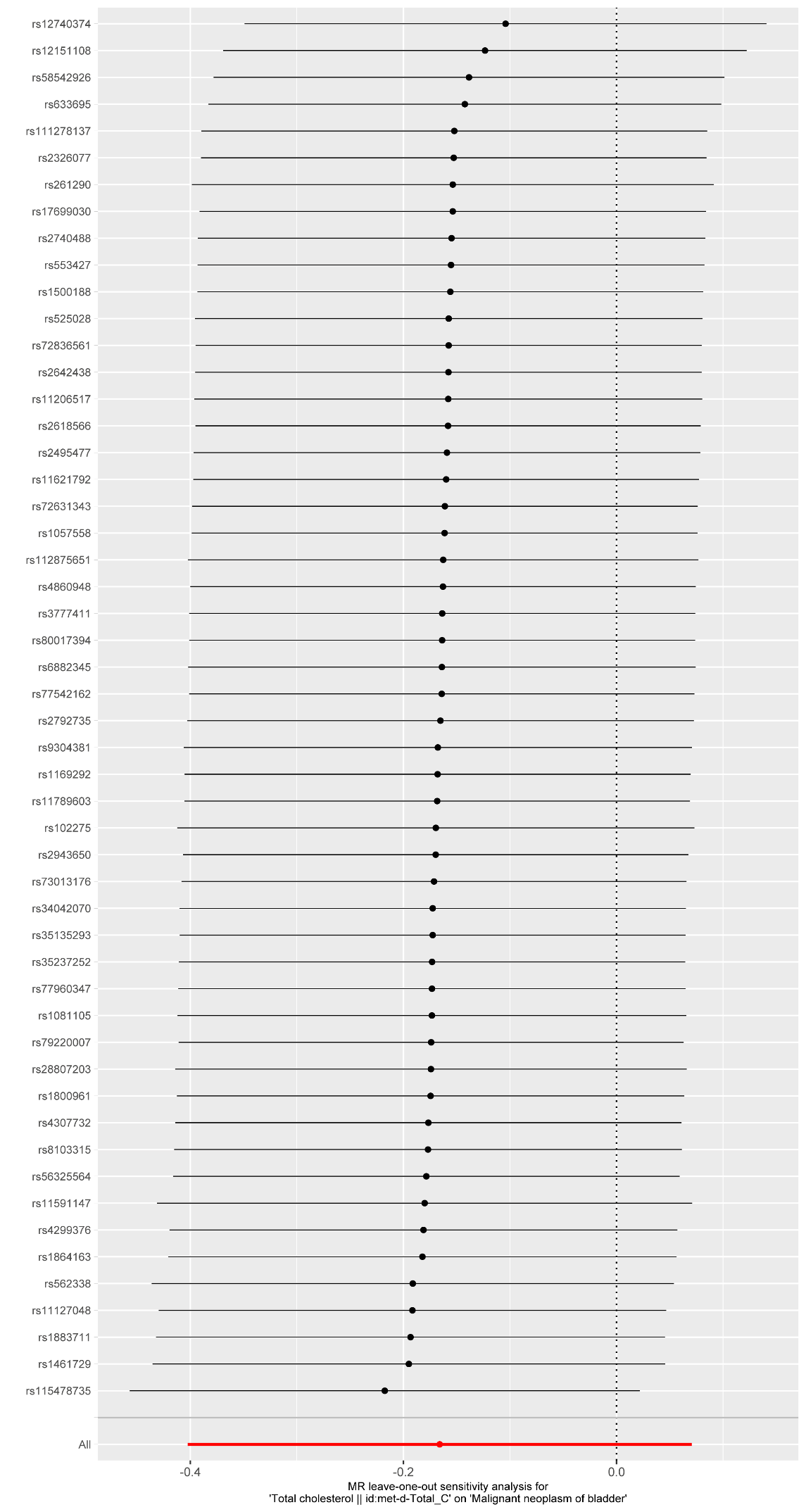


The leave-one-out plot of TG


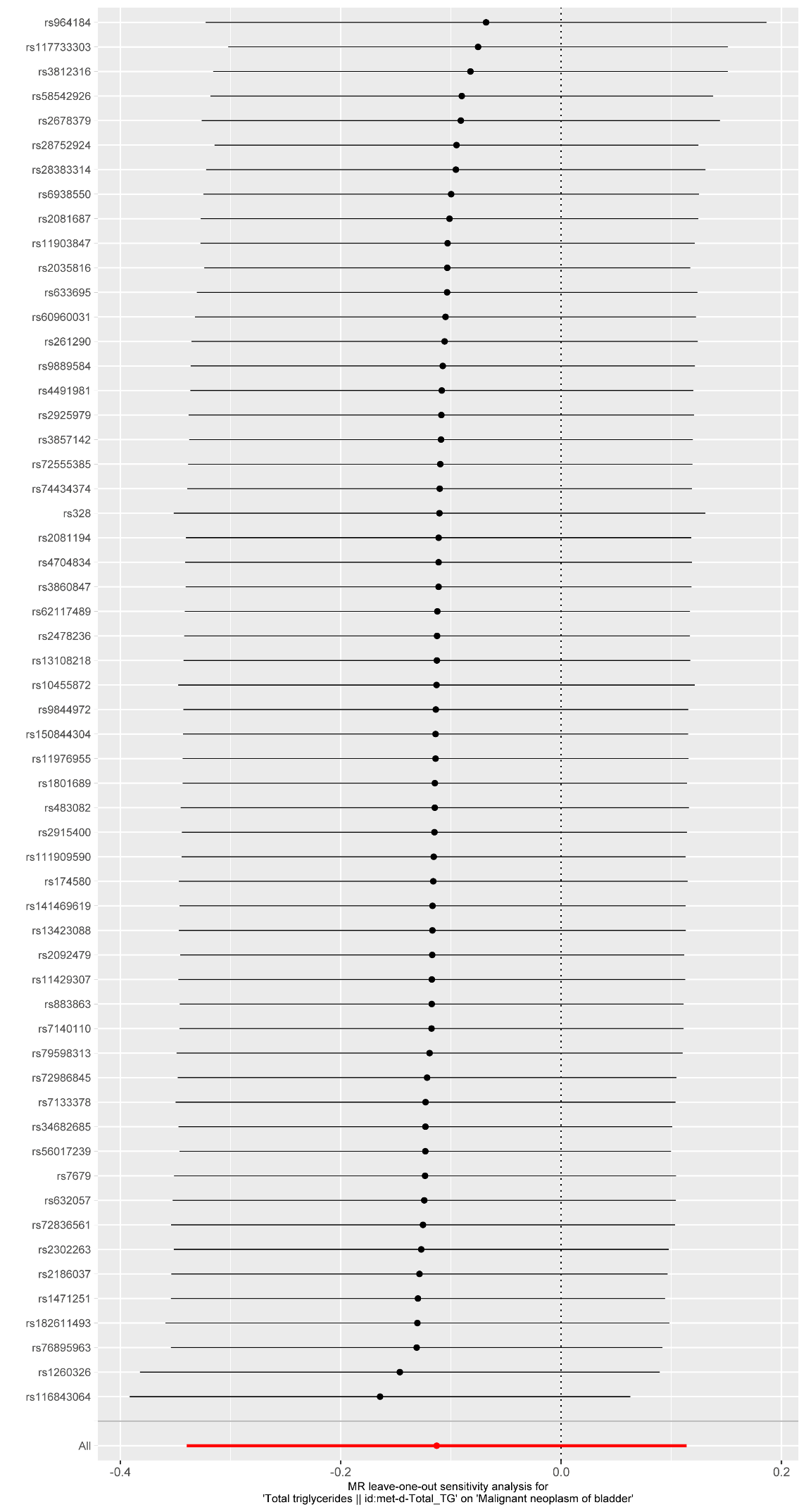

Supplement: Supplementary file 5 [file Table_3.DOCX]
